# Polyglycine Acts as a Rejection Signal for Protein Transport at the Chloroplast Envelope

**DOI:** 10.1371/journal.pone.0167802

**Published:** 2016-12-09

**Authors:** Joshua K. Endow, Agostinho Gomes Rocha, Amy J. Baldwin, Rebecca L. Roston, Toshio Yamaguchi, Hironari Kamikubo, Kentaro Inoue

**Affiliations:** 1 Department of Plant Sciences, University of California at Davis, One Shields Avenue, Davis, California, United States of America; 2 Graduate School of Materials Science, Nara Institute of Science and Technology, Takayama, Ikoma, Nara, Japan; Agriculture and Agri-Food Canada, CANADA

## Abstract

PolyGly is present in many proteins in various organisms. One example is found in a transmembrane β-barrel protein, translocon at the outer-envelope-membrane of chloroplasts 75 (Toc75). Toc75 requires its N-terminal extension (t75) for proper localization. t75 comprises signals for chloroplast import (n75) and envelope sorting (c75) in tandem. n75 and c75 are removed by stromal processing peptidase and plastidic type I signal peptidase 1, respectively. PolyGly is present within c75 and its deletion or substitution causes mistargeting of Toc75 to the stroma. Here we have examined the properties of polyGly-dependent protein targeting using two soluble passenger proteins, the mature portion of the small subunit of ribulose-1,5-bisphosphate carboxylase/oxygenase (mSS) and enhanced green fluorescent protein (EGFP). Both t75-mSS and t75-EGFP were imported into isolated chloroplasts and their n75 removed. Resultant c75-mSS was associated with the envelope at the intermembrane space, whereas c75-EGFP was partially exposed outside the envelope. Deletion of polyGly or substitution of tri-Ala for the critical tri-Gly segment within polyGly caused each passenger to be targeted to the stroma. Transient expression of t75-EGFP in *Nicotiana benthamiana* resulted in accumulation of c75-EGFP exposed at the surface of the chloroplast, but the majority of the EGFP passenger was found free in the cytosol with most of its c75 attachment removed. Results of circular dichroism analyses suggest that polyGly within c75 may form an extended conformation, which is disrupted by tri-Ala substitution. These data suggest that polyGly is distinct from a canonical stop-transfer sequence and acts as a rejection signal at the chloroplast inner envelope.

## Introduction

Single amino acid repeats are abundant in various proteins in eukaryotes, and one of the common repeats are those of Gly [1]. Results of sequence analyses indicate that the prevalence of polyGly in mammals may be driven by the pressure towards G+C richness in the third codon position [1], and that polyGly may serve as the Gly reservoir in plants as its abundance is consistent with the Gly content in the entire proteome [2]. Despite this knowledge, however, the biological functions of polyGly and the underlying mechanisms are largely unexplored. Among a few polyGly with demonstrated functions is the one found in the sorting signal for a chloroplast membrane protein called Toc75 [3].

The chloroplast plays an essential role in viability of photosynthetic eukaryotes [4]. It is surrounded by an envelope comprising the outer and inner envelope membranes (OEM and IEM) that plays versatile roles in organelle biogenesis, metabolism, and intracellular communication [5–10]. The majority of proteins localized to the chloroplast envelope are encoded in the nuclear genome. Most IEM proteins are synthesized as a larger precursor with an N-terminal chloroplast import signal called a transit peptide [11]. Transit peptides are necessary and sufficient for protein targeting to and translocation across the chloroplast envelope via the general import machinery known as the translocons at the outer- and inner-envelope-membrane of chloroplasts (TOC and TIC) [12]. These targeting signals are removed by a soluble metallopeptidase called stromal processing peptidase (SPP) in the stroma [13, 14]. Two pathways are known to sort proteins to the IEM during or shortly after their import via the TOC/TIC machinery [11, 15]. The first pathway inserts the protein by a stop-transfer mechanism. Known substrates of this pathway have a single α-helical transmembrane domain (TMD) that acts as an envelope-halting signal [16, 17]. Although not proven, these signals may be transferred laterally from the TIC complex into the IEM lipid bilayers, as in the case of the analogous mechanism in mitochondria [18]. The second IEM-sorting pathway directs the protein to the stroma before targeting it to the IEM. This so-called postimport pathway has been shown to target three integral TIC subunits, Tic110, Tic40, and Tic21 [19–22]. A Ser/Pro-rich domain at the N terminus to the TMD was found to be important for membrane insertion of Tic40 [19] although a similar domain is not obvious in the other two TIC subunits.

Most OEM proteins [5, 23] and a few IEM Proteins [24–26] are encoded in the nucleus as a mature form without a transit peptide. A series of elegant studies have established that a subset of OEM proteins are co-translationally recognized in the cytosol at their TMD and a positively charged flanking region at its C terminus by an ankyrin repeat protein, which directs its client proteins specifically to the chloroplast OEM [27–29]. Insertion of these proteins involves the core TOC component Toc75 [30], which forms a transmembrane β-barrel [31, 32].

**Fig 1:**
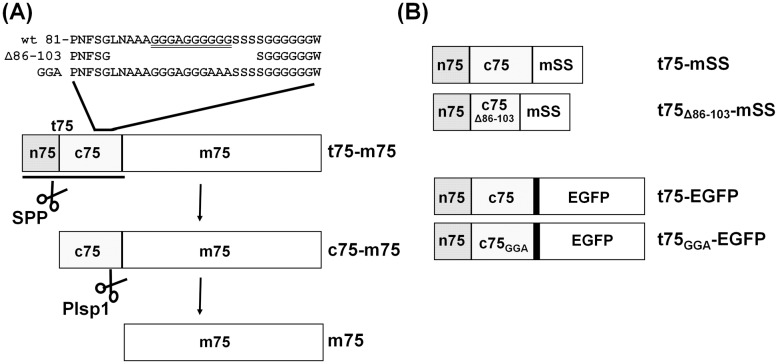
Toc75 and t75-fusion proteins used for the study. (A) Toc75 precursor (t75-m75) carries an N-terminal extension (t75), which comprises n75 and c75, for correct targeting to the chloroplast envelope. Removals of n75 and c75 by SPP and Plsp1 yield the intermediate (c75-m75) and mature form (m75), respectively. Primary sequences of part of c75 and its variants are shown above the diagram. (B) A diagram of four chimeric proteins used in the study.

Toc75 itself is unique among the OEM proteins in that it is synthesized in the cytosol as a larger precursor with an N-terminal extension of 100–140 residues called t75 (also termed tp75) [3, 33]. t75 is required for proper targeting of Toc75 to the OEM and can be divided into n75 and c75 (also termed tpn75 and tpc75, respectively) [3, 34, 35] (Fig 1A). Our current knowledge about t75 is based on results of *in vitro* studies using its ortholog from pea (*Pisum sativum*) known as psToc75, whose n75 and c75 consist of 35 and 96 residues, respectively [33, 35]. n75 acts as a canonical transit peptide and is removed in the stroma [34, 35]. c75 is necessary but not sufficient for targeting Toc75 to the OEM [35] and is removed by membrane-bound plastidic type I signal peptidase 1 (Plsp1) most likely at the intermembrane space (IMS) [36–39]. Within c75 are two conserved regions, a hydrophobic domain (residues 52–77 in psToc75) and a region containing polyGly (residues 91–110 in psToc75) [3] (Fig 1A). By deletion and substitution mutagenesis combined with *in vitro* protein import assay, the N-terminal polyGly corresponding to residues 91–100, but not the hydrophobic domain nor the C-terminal polyGly, was found to be necessary for targeting psToc75 to the OEM [3]. Results of further *in vitro* assays suggested that relatively compact and non-hydrophobic properties of a tri-Gly segment (residues 98–100) within polyGly are crucial for envelope sorting [40]. These results made Toc75 polyGly one of the few examples of this type of a single amino acid repeat with a demonstrated function.

t75 has been used to target two passengers to the chloroplast envelope. One of them is a soluble protein, the mature portion of the small subunit of ribulose-1,5-bisphosphate carboxylase/oxygenase (mSS) from *Nicotiana tabacum*, which was targeted to the chloroplast IMS *in vitro* [35]. Another passenger is a single-pass membrane protein, diacylglycerol kinase from *Escherichia coli*, which was directed to the chloroplast OEM where it manipulated lipid metabolism *in vivo* [41]. However, it had not been tested if polyGly plays any roles in targeting of these passengers, which do not form a transmembrane β-barrel like Toc75. Also unexplored was the mechanism by which c75 mediates protein sorting to the chloroplast envelope. Here, we have used *in vitro* and *in vivo* assays to demonstrate that the requirement of polyGly for c75-mediated envelope-sorting is not limited to a transmembrane β-barrel passenger. Together with the spectroscopic data, our results suggest that the polyGly-dependent envelope sorting is distinct from the canonical stop transfer and that it involves a rejection mechanism at the envelope.

## Materials and Methods

### Preparation of radiolabeled proteins

Radiolabeled proteins examined by *in vitro* import assays were synthesized with plasmids described below using T_N_T^®^ Coupled Reticulocyte Lysate System (Promega, Madison, WI, USA), T7 (for Tic22, Tic40, and t75-mSS variants), SP6 (for t75-EGFP variants), or T3 (for DGD1) RNA polymerase, and [^35^S]Met (PerkinElmer Life Sciences, Boston, MA, USA) according to the manufacturer’s instructions. The plasmids encoding Tic22 [42], Tic40 [19], and DGD1 [43] were used to generate control proteins of known localization. Plasmids for t75-mSS variants were generated using pET23-prSS (precursor of pea SS in a pET23 vector) [34], whose digestion with *Nco*I and *Sph*I conveniently removes the transit peptide-coding sequence and yields a 4-kb fragment including a region encoding mSS. The DNA sequence for residues 1–136 of psToc75, which contains the entire t75 portion of 131 residues and five N-terminal residues of the mature portion, was amplified using pET23-pr75 [34] as a template and primers carrying *Nco*I or *Sph*I site. After digestion with *Nco*I and *Sph*I, the PCR product was ligated into the 4-kb *Nco*I-*Sph*I fragment from pET23-prSS, yielding pET23-t75-mSS (S7 Fig). For the DNA sequence encoding t75_Δ86–103_, 5’- and 3’-fragments encoding residues 1–85 and 100–125 of psToc75, respectively, were amplified using pET23-pr75 as a template and a set of primers, each of which contained *Nde*I or *Bam*HI site (for 5’-fragment) or *Bam*HI or *Eco*RI site (for 3’-fragment), respectively. The obtained fragments were digested with *Nde*I and *Bam*HI, and *Bam*HI and *Eco*RI, respectively, and ligated together with the 5.7-kb *Nde*I-*Eco*RI fragment of pET23-pr75. The resultant plasmid named pET23-pr75_Δ86–103_ was used as a template to amplify the t75_Δ86-103_-coding sequence, which was ligated into the pET23-t75_Δ86-103_-mSS plasmid as described above for pET23-t75-mSS. For the t75-EGFP variants, the sequences encoding t75 and t75_GGA_ (including 10 N-terminal residues of mature psToc75) were amplified using pET23-pr75 and pET23-pr75_GGA_ [40], respectively, and a set of primers, each of which carried *Spe*I or *Bgl*II site. Each of the obtained PCR products was digested with *Spe*I and *Bgl*II and ligated individually into the *Spe*I-*Bgl*II site of pB-CG, a binary plasmid that contains Cauliflower Mosaic Virus 35S promoter followed by a sequence encoding a linker of 17 amino acids and EGFP (S8 Fig). The resulting plasmids named pB-CG-t75 and pB-CG-t75_GGA_ were used as templates to amplify sequences encoding t75-EGFP and t75_GGA_-EGFP, respectively (S1 Fig). The obtained sequences were subcloned individually into a pGEM^®^-T Easy vector (Promega, Madison, WI, USA). All the primers used for PCRs are listed in S1 Table, and the identity of the obtained constructs was confirmed by sequencing.

### *In vitro* chloroplast protein import

Pea chloroplast preparation and *in vitro* protein import assay were performed as described [3]. Briefly, chloroplasts isolated from pea seedlings (Little Marvel from Seedway, Elizabethtown, PA, USA) grown on vermiculite at 20–23°C with 12 h light (60 μmol/m^2^·s) per day for 11–14 days were incubated with radiolabeled precursors described above under light at room temperature for 30 min. Separation of chloroplasts containing imported proteins into various fractions was done as described previously [44]. In brief, isolated chloroplasts were resuspended with hypotonic lysis buffer (10 mM Hepes, pH 8.0, 10 mM MgCl_2_) and separated into supernatant (S1) and pellet fractions by centrifugation at 16,000 *g*, 4°C for 20 min. The resultant pellet was resuspended in 0.1 M Na_2_CO_3_, centrifuged again at 16,000 *g*, 4°C for 20 min, and separated into the second supernatant (S2), which contained peripheral membrane proteins, and the final pellet (P), which included integral membrane proteins. Post-import protease treatment was done as described [45] with some modifications. Thermolysin treatment included 1 μg thermolysin (P1512 from Sigma-Aldrich, St. Louis, MO, USA) per μg chlorophyll equivalent chloroplasts in a final concentration of 0.5 μg/μl chlorophyll in import buffer containing 1 mM CaCl_2_. The reaction was performed on ice in the dark for 30 min and terminated by addition of an equal volume of import buffer containing 20 mM EDTA. Buffers used for subsequent lysis and wash processes contained 5 mM EDTA. Trypsin treatment included 0.5 μg trypsin (T1426 from Sigma-Aldrich) per μg chlorophyll equivalent chloroplasts in a final concentration of 0.5 μg/μl chlorophyll in import buffer. The reaction mixture was incubated for 60 min at room temperature in the dark, and the protease activity was quenched by addition of 10 μg trypsin inhibitor (T9003 from Sigma-Aldrich) per μg trypsin. All buffers used for subsequent processes included 0.05 μg/μl trypsin inhibitor. For both treatments, the protease activity was controlled by performing a reaction with the presence of 1% (v/v) Triton X-100 followed by 10% (w/v) TCA precipitation. Processing by bacterially-produced Plsp1_Δ2–67_ was performed at room temperature for 2 h as described [36]. All the products of the import and processing assays were separated by SDS-PAGE and visualized using a phosphorimager. Quantification of radioactive signals was done using ImageJ 1.48v (National Institute of Health). The protein gels including the radiolabeled products were also stained with Coomassie Brilliant Blue where indicated.

### Transient expression

For transient expression in *N*. *benthamiana*, pMDC32 carrying the coding sequence of Plsp1 [46] was used for the non-fluorescent control, and two binary plasmids described above, pB-CG-t75 and pB-CG-t75_GGA_, were used for the production of t75-EGFP and t75_GGA_-EGFP proteins, respectively. *Agrobacterium tumefaciens* GV3101 cells carrying each of these plasmids were grown overnight in 2 ml of 50 mg/l kanamycin, 25 mg/l gentamycin, and 17 mg/l rifampicin in lysogeny broth (LB). Cells were then diluted with 40 ml of LB containing 50 mg/l kanamycin and incubated at 28°C until OD600 reached 0.2. Cells were harvested by centrifugation at 3,000 *g*, 4°C for 10 min and resuspended in 16 ml of induction medium containing 10 mM MES-KOH, pH 5.6, 1 mM MgCl_2_, 0.2% (w/v) glucose, and 0.15 mM acetosyringone. Cells were then incubated further at 120 rpm, 28°C for 2 h, and harvested by centrifugation at 3,000 *g*, 4°C for 10 min. The resultant cell pellets were resuspended up to an OD600 of 0.5 with an infiltration medium containing 5% (w/v) sucrose and 0.3 mM acetosyringone, and were infiltrated into the epidermal cells of six- to eight-week-old *N*. *benthamiana* leaves using a syringe. The infiltrated plants were kept in the dark for 14–18 h, transferred to 16-h light (50–80 μmol/m^2^·s) per day at 23–25°C for two to three days before analysis. Chloroplasts were isolated from infiltrated regions of leaves using a grinding method developed for Arabidopsis [21]. Subsequent analyses were performed as described above for chloroplast import except that the protein detection was done by immunoblotting using antibodies against GFP (Santa Cruz Biotechnology, CA, USA) and Coomassie Brilliant Blue staining where indicated. Confocal microscopy was performed with a LSM 710 AxioObserver (Carl Zeiss, Oberkochen, Germany) with an LD C-Apochromat 40×/1.1 W Korr M27 water immersion objective using excitation with the 488 nm laser line and emission detected for GFP from 495 nm to 545 nm and chlorophyll from 655 nm to 705 nm. Acquisition and subsequent linear adjustments of the signal intensities of all images at a given zoom level were performed at the identical settings.

### Circular dichroism (CD) analysis

Two peptides of 24 amino acids, t75_89-112_ (Ala-Ala-Gly-Gly-Gly-Ala-Gly-Gly-Gly-Gly-Gly-Gly-Ser-Ser-Ser-Ser-Gly-Gly-Gly-Gly-Gly-Gly-Trp-Phe) and t75_GGA89-112_ (Ala-Ala-Gly-Gly-Gly-Ala-Gly-Gly-Gly-Ala-Ala-Ala-Ser-Ser-Ser-Ser-Gly-Gly-Gly-Gly-Gly-Gly-Trp-Phe), were purchased from EZBiolab (Carnel, IN, USA) with purify of 95%, dissolved to 141 μM and 99.4 μM, respectively, in 8.5 mM Tris-HCl, pH 7.5 and 10% (v/v) hexafluoroisopropanol, and used for the analysis. CD spectra were measured at 20°C with a J-725 circular dichroism spectropolarimeter (Jasco, Tokyo, Japan) using a 0.1-cm path-length quartz cell.

## Results

### t75 targets soluble passenger proteins to the chloroplast envelope in a polyGly-dependent manner *in vitro*

**Fig 2:**
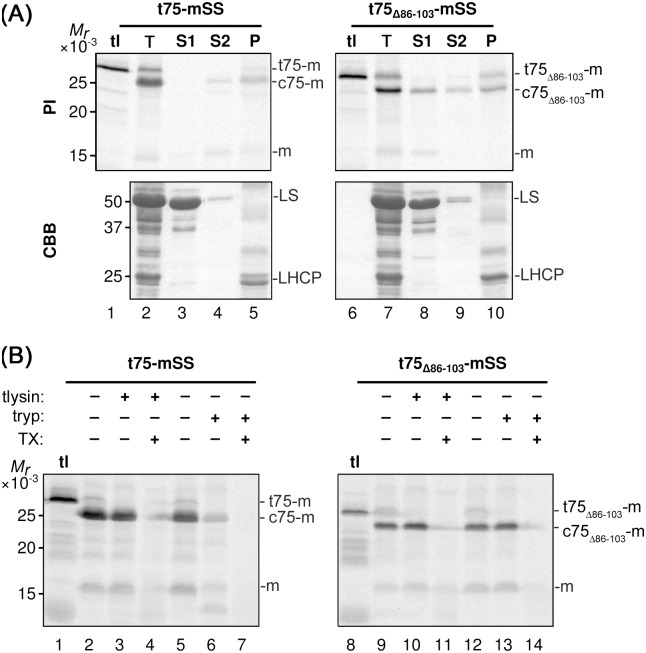
*In vitro* import of t75-mSS variants. (A) Radiolabeled t75-mSS variants indicated above were incubated with isolated chloroplasts under the import condition. After 30-min of import, intact chloroplasts were reisolated and separated into two aliquots. The first aliquot was kept as total chloroplasts (T). The second aliquot was hypotonically lysed and fractionated by centrifugation to a supernatant (S1) and the pellet. The pellet was then resuspended with 0.1M Na_2_CO_3_ and fractionated by centrifugation to the second supernatant (S2) and the final pellet fraction (P). Samples equivalent to 3 μg chlorophyll were separated by SDS-PAGE, and radiolabeled proteins and total proteins in each sample were visualized by phosphorimaging (PI) and Coomassie Brilliant Blue staining (CBB), respectively. The experiments were done concurrently with those shown in Fig 3A and S1 Fig. tl contained the translation product corresponding to the one used for the import assay with 3 μg chlorophyll-equivalent chloroplasts. The precursors containing the entire t75 variants, the intermediates that carrying the c75 variants, and the mature forms lacking the entire t75 variant, respectively, are indicated at right; mSS is indicated with the letter m. For the CBB panel, large subunit of ribulose-1,5-bisphosphate carboxylase/oxygenase and light-harvesting chlorophyll a/b-binding protein are indicated as LS and LHCP, respectively. (B) After the import reaction as described in the legend to panel (A), intact chloroplasts were reisolated and separated into six aliquots. Three of them were resuspended in import buffer containing 1 mM CaCl_2_ with or without 1 μg thermolysin (tlysin) per μg chlorophyll equivalent chloroplasts and 1% Triton X-100 (TX) as indicated, incubated for 30 min on ice in the dark. Other three aliquots were resuspended in import buffer with or without 0.5 μg trypsin (tryp) per μg chlorophyll equivalent chloroplasts and 1% Triton X-100 (TX) as indicated, incubated for 60 min at room temperature in the dark. The activities of thermolysin and trypsin were quenched by 10 mM EDTA and 10 μg trypsin inhibitor per μg trypsin, respectively. Samples equivalent to 3 μg chlorophyll were separated by SDS-PAGE and radiolabeled proteins visualized by phosphorimaging. The experiments were done concurrently with those shown in Fig 3B and S2 Fig. For the labels at right, see the legend to panel (A).

As a first step to test the role of polyGly in t75-dependent targeting of non-native passengers, t75 and its variant lacking polyGly (t75_Δ86–103_) (Fig 1B) were independently fused to mSS and subjected to import assay using chloroplasts isolated from pea seedlings. The main import product from t75-mSS and t75_Δ86-103_-mSS migrated on SDS-PAGE around 25 kD and 24 kD, respectively, corresponding to the size lacking n75 but retaining c75 (Fig 2A, panel PI, indicated as c75-m and c75_Δ86-103_-m, respectively). By fractionation, an endogenous soluble protein, the large subunit of ribulose-1,5-bisphosphate carboxylase/oxygenase (LS), was recovered in the S1 fraction (Fig 2A, panel CBB, lanes 3 and 8), an endogenous integral membrane protein, light-harvesting chlorophyll a/b-binding protein (LHCP), was found in the P fraction (Fig 2A, panel CBB, lanes 5 and 10), and a peripheral membrane protein Tic22 was targeted to the S2 and P fractions (S1 Fig) as reported previously [47]. Under these conditions, c75-mSS was found predominantly in the S2 and P fractions (Fig 2A, panel PI, lanes 4 and 5) similar to the case with Tic22. By contrast, c75_Δ86-100_-mSS was recovered in all three fractions (Fig 2A, panel PI, lanes 8–10). To determine suborganellar location of the imported proteins, intact chloroplasts were reisolated and treated with thermolysin, which cannot pass through the OEM and thus can only access proteins at the outer surface of the OEM [48], or trypsin, which can pass through the OEM but not the IEM thus can reach proteins located at the OEM, IMS, and the outer leaflet of the IEM [49]. Under the conditions used, the imported IEM protein, Tic40, was resistant to both proteases, while the IMS protein, Tic22, was largely degraded by trypsin but not by thermolysin (S2 Fig). Consistent with the previous result [35], c75-mSS was largely resistant to thermolysin (with 10% degradation as judged by quantification using ImageJ software) and susceptible to trypsin (Fig 2B, compare lanes 2 and 3, 5 and 6). By contrast, c75_Δ86-103_-mSS was resistant to both proteases unless a detergent was present to disrupt the lipid bilayers (Fig 2B, lanes 9–14). These results demonstrate that deletion of polyGly disrupts t75-dependent targeting of a soluble passenger to the chloroplast envelope, i.e., turning t75 to the chloroplast-stroma targeting signal, and that complete removal of t75 is not required for stroma localization, similar to the case with Toc75 [3].

**Fig 3:**
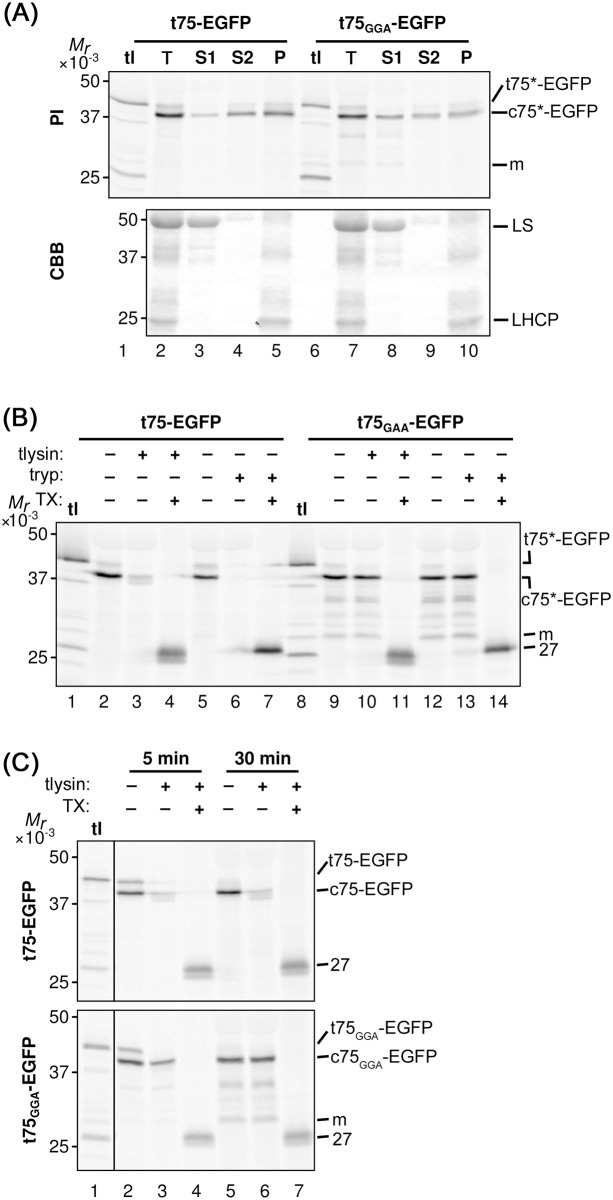
*In vitro* import of t75-EGFP variants. (A) Import of radiolabeled t75-EGFP variants into isolated chloroplasts, post-import fractionation, and analysis of the results were done as described in Fig 2A. The experiments were done concurrently with those shown in Fig 2A and S1 Fig. The precursor proteins containing the entire t75 variants, the intermediates containing the c75 variants but not n75, and the mature form that lacks the entire t75 variants are indicated as t75*-EGFP, c75*-EGFP, and m, respectively. For the CBB panel, large subunit of ribulose-1,5-bisphosphate carboxylase/oxygenase and light-harvesting chlorophyll a/b-binding protein are indicated as LS and LHCP, respectively. (B) Import of radiolabeled t75-EGFP variants into isolated chloroplasts, post-import protease treatment, and analysis of the results were done as described in Fig 2B. The experiments were done concurrently with those shown in Fig 2B and S2 Fig. The 27-kD protease-protected EGFP is indicated as 27. For other labels, see the legend to panel (A). (C) Import of radiolabeled t75-EGFP variants into isolated chloroplasts were performed for 5 or 30 min followed by post-import treatment with thermolysin as described in Fig 2B. The precursor proteins containing the entire t75 variants, the intermediates containing the c75 variants but not n75, and the mature form that lacks the entire t75 variants are indicated as t75-EGFP/t75_GGA_-EGFP, c75-EGFP/c75_GGA_-EGFP, and m, respectively. The 27-kD protease-protected EGFP is indicated as 27.

To further confirm the requirement of polyGly for t75-dependent protein targeting to the chloroplast envelope *in vitro* and also to prepare for testing the function of polyGly for *in vivo* targeting, we examined enhanced green fluorescent protein (EGFP) as the second soluble passenger. EGFP is a useful reporter for *in vivo* targeting because it is foreign to chloroplasts, eliminating the interference by the endogenous proteins, and its localization can be examined by microscopy [50]. A previous study showed that replacement of the tri-Gly segment at positions 98 to 100 with tri-Ala, which was named the GGA mutation as it retains the preceding two tri-Gly segments at positions 91 to 93 and 95 to 97, respectively, caused mis-localization of pea Toc75 to the stroma [40], similar to the case with the polyGly deletions [3]. Thus, we prepared constructs encoding t75_GGA_-EGFP in addition to the non-mutated form. For both t75-EGFP variants, after a 30-min import reaction, the major protein of 38 kD, which corresponds to the protein that lacks n75 but still retains c75, was distributed to all the three fractions (Fig 3A, panel PI, indicated as c75*-EGFP). Interestingly, 66% of imported c75-EGFP, as judged by quantification using ImageJ, was degraded by thermolysin (Fig 3B, compare lanes 2 and 3), in contrast to the case with c75-mSS, which showed only 10% degradation (Fig 2B, compare lanes 2 and 3). Note that with the presence of detergent, the proteolysis assay resulted in the formation of a 27-kD band (indicated as 27 in Fig 3B, lanes 4, 7, 11, and 14). It is known that treatments with various detergents including Triton X-100 do not affect folding of GFP [51], and that properly folded GFP is resistant to proteases [52]. Thus, the result suggests that the detergent caused the conformational change of imported EGFP from a protease-susceptible form to a protease-resistant folded form. The thermolysin-susceptibility of c75-EGFP was consistent after 5-min and 30-min import reactions (Fig 3C, compare lanes 2 and 3, 5 and 6) and also after various incubation times with thermolysin (S3 Fig). Trypsin-sensitivity of imported c75-EGFP was, however, similar to the case with c75-mSS, which was largely digested (lanes 5 and 6 in Figs 2B and 3B). By contrast, imported c75_GGA_-EGFP was largely resistant to both proteases unless the detergent was present in the reaction (Fig 3B, lanes 9–14), similar to the case with c75_Δ86-103_-mSS (Fig 2B, lanes 9–14). These data indicate that a significant amount of imported c75-EGFP was exposed to the outer surface of the chloroplast OEM, while c75_GGA_-EGFP was mainly localized to the stroma.

Together, these results show that polyGly within c75 is necessary for preventing soluble passenger proteins from traversing the IEM to the stroma. The final location appears to depend on each passenger protein, i.e., mSS in the IMS and EGFP exposed to the surface of the OEM.

### PolyGly is necessary for preventing protein translocation across the chloroplast envelope membrane *in vivo*

**Fig 4:**
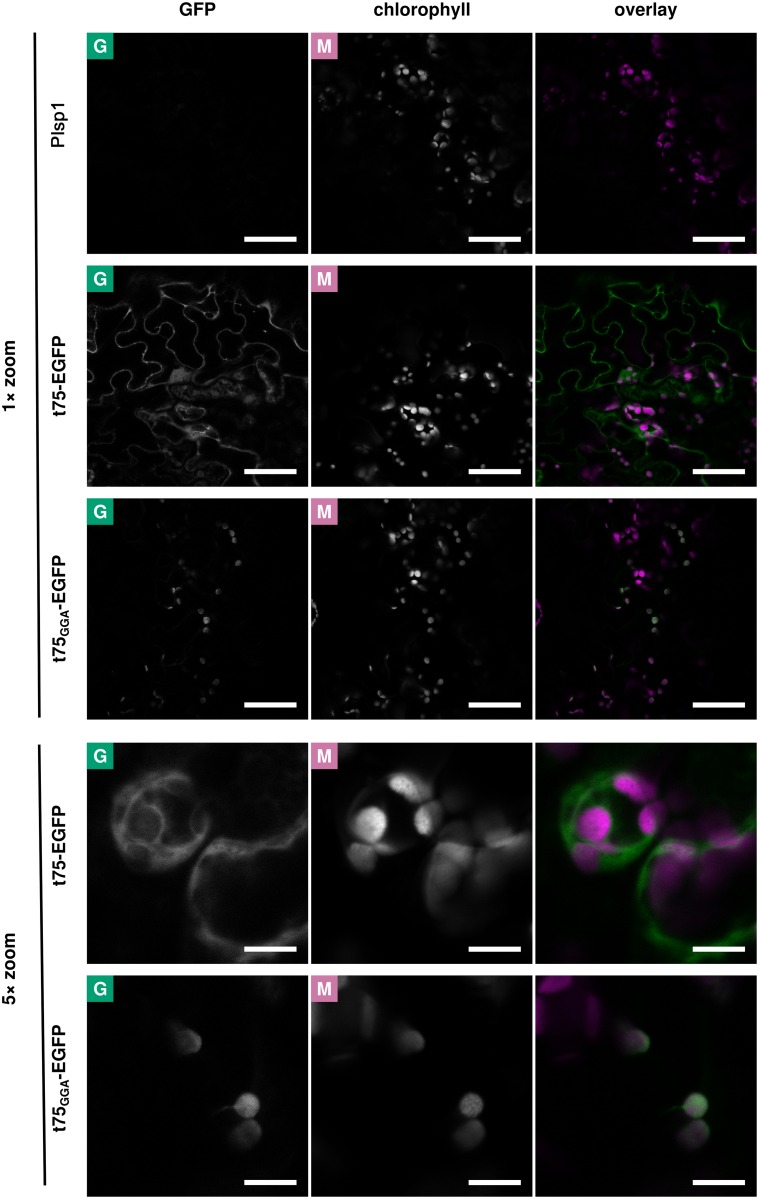
Confocal microscopy analysis of t75-EGFP variants after transient expression in *N*. *benthamiana*. Leaves of *N*. *benthamiana* were agroinfiltrated with constructs encoding Plsp1 (non-fluorescent control), t75-EGFP, or t75_GGA_-EGFP. Fluorescence from EGFP and chlorophyll was observed in infiltrated leaves by confocal microscopy. In the overlay, signals from the GFP and chlorophyll channels are depicted as green and magenta, respectively. Scale bars represent 50 μm (1× zoom) or 10 μm (5× zoom). See S4–S6 Figs for additional sets of images.

The localization of c75-EGFP detected after *in vitro* import might represent an import intermediate due to limitations of the assay, such as the lack of components needed to complete targeting and processing of t75-EGFP. To address this possibility, we conducted a transient expression assay using *Nicotiana benthamiana* leaf cells. Similar to the case with *in vitro* assay, t75_GGA_-EGFP was included as a control. Also expression of a non-fluorescent protein (Plsp1) was performed to control background signal (Fig 4 and S4 Fig, panel Plsp1). Two to three days after infiltration, the signal derived from t75-EGFP was mostly present in the cytosol; only occasionally it was found adjacent to the chlorophyll signal (Fig 4 and S4 Fig, panel t75-EGFP; S5 Fig). By contrast, GFP signals in t75_GGA_-EGFP-expressing cells were co-localized mainly with the chlorophyll autofluorescence (Fig 4 and S4 Fig, panel t75_GGA_-EGFP; S6 Fig).

**Fig 5:**
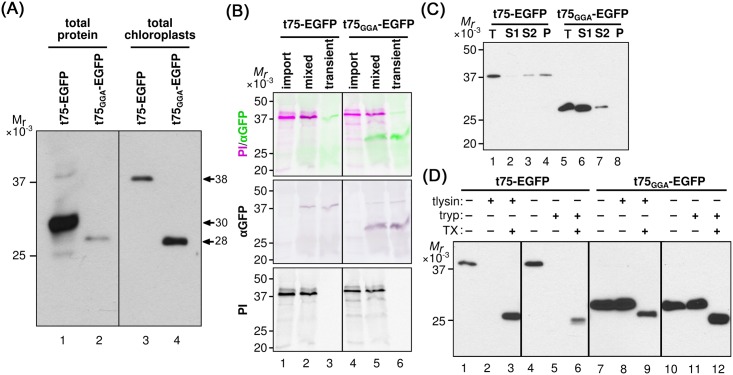
Biochemical analysis of t75-EGFP variants after transient expression in *N*. *benthamiana*. (A) Total protein extracts equivalent to *ca*. 12.5 mg fresh weight and total chloroplasts containing 10 μg chlorophyll prepared from leaves expressing t75-EGFP or t75_GGA_-EGFP were separated by SDS-PAGE and analyzed by immunoblotting with the anti-GFP antibody. The bands corresponding to proteins of 38 kD, 30 kD, and 28 kD are indicated. Different regions from the same membrane are separated by a black line. (B) Total chloroplasts after import (import) as in Fig 3A, total chloroplasts from agroinfiltrated leaves (transient) as in panel (A), and a mixture of them were separated by SDS-PAGE and detected by immunoblotting with the anti-GFP antisera (αGFP) and phosphorimaging (PI). The combined image is false-colored green for αGFP and magenta for PI. Different regions from the same membrane are separated by black lines. Import samples were loaded at 2 μg chlorophyll equivalents and transient expression samples were loaded at 10 μg chlorophyll equivalents. (C) Ten μg chlorophyll equivalent total chloroplasts (T) isolated from *N*. *benthamiana* leaves expressing t75-EGFP or t75_GGA_-EGFP as well as S1, S2, and P fractions as prepared in Fig 2A were separated by SDS-PAGE and proteins were detected by immunoblotting with the antisera against GFP. (D) Intact chloroplasts isolated from *N*. *benthamiana* leaves expressing t75-EGFP or t75_GGA_-EGFP were incubated with thermolysin (tlysin) or trypsin (tryp) with or without the presence of Triton X-100 (TX) as described in the legend to Fig 2B. Loading and detection were performed as in panel (C).

To further characterize the properties of the EGFP variants produced by the transient assay, total protein extract and intact chloroplasts were prepared from the *N*. *benthamiana* leaves and analyzed by immunoblotting using the anti-GFP antibody. As shown in Fig 5A, the main immunoreactive bands in the total extract from t75-EGFP-expressing leaves migrated around 30 kD (lane 1), which was not detected in the total chloroplasts (lane 3); the only band detected in the chloroplasts migrated around 38 kD (lane 3), which was also detectable in the total extracts but its intensity was much lower than that of the 30-kD band (lane 1). This result indicates that the GFP signals found in the cytosol and chloroplasts under the microscope in t75-GFP-expressing plants (Fig 4 and S4 Fig, panel t75-EGFP; S5 Fig) were derived from two distinct proteins of 30 kD and 38 kD, respectively. By contrast, in the case of t75_GGA_-EGFP-expressing plants, the immunoreactive band in the total extract migrated around 28 kD (Fig 5A, lane 2), corresponding to the band found in the chloroplast fraction (Fig 5A, lane 4). These data support that the 28-kD protein in the total extracts in t75_GGA_-EGFP-expressing leaves represents the chloroplast-localized GFP signal (Fig 4 and S4 Fig, panel t75_GGA_-EGFP; S6 Fig).

For t75-EGFP, the immunoreactive 38-kD band found in the chloroplast of the transformed plants behaved similarly to the main product of *in vitro* import assay in not only the mobility on SDS-PAGE (Fig 5B, lanes 1–3) but also the distribution to the peripheral (S2) and integral (P) membrane fractions (Fig 5C, lanes 3 and 4) and the high susceptibility to both thermolysin and trypsin (Fig 5D, compare lanes 1 and 2, 4 and 6). Note in Fig 5B lanes 1, 2, 4, and 5 that the amount of the imported radiolabeled protein was sufficient to be detected by phosphorimager (panel PI) but not by immunoblotting using the anti-GFP antibody (panel αGFP). Also note that as in the case of *in vitro* import assay, with the presence of detergent, the proteolysis of EGFP variants yielded a 27-kD band, which corresponded to protease-protected EGFP (Fig 5D, lanes 3 and 6). By contrast, for t75_GGA_-EGFP, the immunoreactive protein in the chloroplast generated by the transient expression assay migrated faster than c75_GGA_-EGFP produced by *in vitro* import (Fig 5B, lanes 4–6). Indeed, the size of this protein (*ca*. 28 kD) corresponds to that of the protein lacking the entire t75_GGA_ portion from t75_GGA_-EGFP. By fractionation and protease-treatment of chloroplasts from the t75_GGA_-EGFP-expressing plants, the 28-kD protein was mainly recovered in the S1 fraction (Fig 5C, lane 6) and resistant to both proteases (Fig 5D, lanes 7–12), indicating its stroma localization.

Results of the transient assay provide further support for the idea that PolyGly is required for preventing protein translocation across the envelope to the stroma. However, some of the results were inconsistent with those of the *in vitro* import. In the case of t75-EGFP, the proteins targeted to the chloroplast showed a similar localization pattern by *in vitro* import (Fig 3A and 3B) and transient expression (Fig 5C and 5D). However, the transient assay revealed the presence of a significant amount of the 30-kD protein outside the chloroplast (Figs 4 and 5A), which could not be detected by the *in vitro* import assay. The GFP signals detected by confocal microscopy should be derived from a folded protein with its C terminus mostly intact [53–55]. Removal of n75 or that of entire t75 from t75-EGFP yield proteins of 38 kD or 28 kD, respectively. Thus, the cytosolic 30-kD protein derived from the t75-EGFP should retain the C-terminal GFP portion and lack the majority but not the entire part of c75. In the case of t75_GGA_-EGFP, by contrast, the *in vitro* import assay generated an apparent import intermediate which still carried the c75_GGA_ and associated with the membrane, while in the transient expression assay, c75_GGA_ was completely removed and the resultant EGFP passenger was released as a soluble protein in the stroma. These data suggest that c75 may promote membrane association independently of PolyGly-mediated envelope sorting.

### PolyGly does not affect processing of c75 by Plsp1 *in vitro*

**Fig 6:**
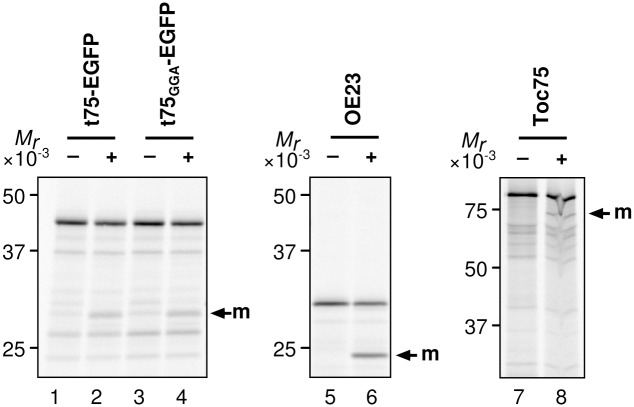
Processing of t75-EGFP variants by Plsp1 *in vitro*. Radiolabeled proteins indicated at top were incubated without or with recombinant Plsp1 protein for 2 h at room temperature, separated by SDS-PAGE, and detected by phosphorimaging.

Import of t75-EGFP into chloroplasts by both *in vitro* and *in vivo* assays as well as import of t75_GGA_-EGFP into isolated chloroplasts resulted in removal of n75 but not that of c75 (Figs 3 and 5), while c75 was completely removed from t75_GGA_-EGFP *in vivo* (Fig 5). These results indicate that the c75 removal by Plsp1 is limiting in the *in vitro* assay as was suggested previously [33]. The data also suggest that PolyGly may prevent removal of c75 by Plsp1. To address this possibility, we examined the processing activity of recombinant Plsp1 against the t75-EGFP variants *in vitro*. The activity was controlled using known Plsp1 substrates, OE23 and Toc75 [36] (Fig 6, lanes 5–8). As shown in Fig 6, both t75-EGFP variants were processed to a protein of *ca*. 28 kD, the size of the protein lacking the entire t75 portion (lanes 2 and 4). This result indicates that, under the conditions used, polyGly did not affect processing of c75 *in vitro*.

### PolyGly can have an extended conformation

**Fig 7:**
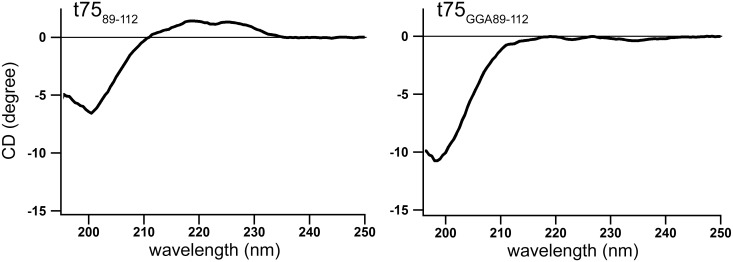
Far UV CD spectra t75_89-112_ variants at 20°C. Each spectrum is the average of eight measurements.

Canonical stop-transfer signals found in chloroplast IEM proteins utilize a hydrophobic segment [16, 17], similar to those found in the mitochondria [18]. By contrast, results of import assays indicate that polyGly within c75 is needed for envelope sorting but is dispensable for association with the membrane (Fig 3A). Thus, polyGly-mediated envelope sorting appears to be distinct from canonical stop transfer. To gain further insight into the mechanism of the polyGly-dependent envelope sorting, peptides corresponding to residues 89 to 112 of t75 and t75_GGA_ were synthesized and their properties examined by circular dichroism (CD) analysis at 20°C. As shown in Fig 7, t75_89-112_ gave a large negative band at 200 nm as well as a weak positive area at 220 nm, while t75_GGA89-112_ showed only a negative band at 200 nm. This result suggests that the two peptides have distinct conformations. The positive peak at 220 nm is one of the characteristics of a left-hand extended helix, such as poly(Pro)II helix. Indeed, the CD spectrum of t75_89-112_ is very similar to that of poly(Pro)II, which exhibits a large negative peak at 200 nm accompanied by a broad positive peak around 220 nm [56]. Furthermore, a previous structural analysis using nuclear magnetic resonance and small-angle X-ray scattering revealed that polyGly can also form an elongated structure similar to poly(Pro)II in solution [57]. These data suggest that t75_89-112_ has an extended poly(Pro)II-like structure although it comprises polyGly instead of polyPro. By contrast, the spectrum of t75_GGA89-112_ is similar to that of a random coil [58], indicating that the replacement of tri-Gly by tri-Ala disrupts the extended structure of t75_89-112_. The structure of such short peptides may not be exactly the same as that in the context of the full protein. Nonetheless the obtained data suggest that polyGly within t75 may form a distinct conformation, and its disruption may abolish the envelope-sorting activity.

## Discussion

PolyGly is one of the common types of single-amino acid repeats [1]. It is present in various proteins such as Class III POU transcription factors [59], the eukaryotic translation release factor 3 [60], and a basic helix loop helix protein associated to schizophrenia [61] in mammals. The importance of polyGly in the protein functionality has been reported in some cases [61–63] although its underlying mechanism is largely elusive. The results presented in the current study demonstrate that polyGly within the Toc75-sorting signal prevents the passenger, regardless of its properties, from entering the chloroplast stroma. Although the envelope-sorting function of polyGly appears unique to that in Toc75, its sensitivity to Ala substitution (Figs 3–5) and potentially extended conformation (Fig 7) demonstrated in this study may be shared with polyGly in other proteins. Thus, our findings may help examine the function of this type of single amino acid repeat, which is present in many proteins in various organisms.

Together with the previous data, our results show that t75 can act as a signal to direct various passenger proteins to the chloroplast envelope in a polyGly-dependent manner. The final localization of the passenger depends on its properties. If the passenger is soluble and c75 is still attached, it may stay associated to the membrane, which can be either IEM or OEM. After import into isolated chloroplasts, c75-mSS was found in the IMS (Fig 2), while c75-EGFP was partially exposed to the outer surface of the OEM (Fig 3). The lack of n75 and presence of c75 indicate that the N termini of both proteins were processed in the stroma but their C termini were rejected at the IEM before it reached Plsp1. mSS is a native chloroplast stroma protein [64]. By contrast, EGFP is a foreign protein although it can be imported into chloroplasts [50]. The difference of their final location may be due to their distinct properties, such as folding, which may be optimized for chloroplast-localization in the case of mSS (thus it remains in the IMS) but not in the case of EGFP (thus it is localized outside). Examining other soluble passengers, both native and foreign to chloroplasts, should address this possibility. If the passenger forms a transmembrane β-barrel like Toc75, it is integrated into the OEM [35]. The folding and membrane integration may be catalyzed by machinery similar to the ones evolutionary conserved in bacteria and mitochondria which may include Toc75 itself or its paralog called outer envelope protein 80 [65, 66]. It is worth mentioning that polyGly may also play a role in targeting α-helical transmembrane proteins to the IEM, as was suggested for P-type ATPase of Arabidopsis 1 [67, 68]. The results of the CD analysis suggest that polyGly may form an extended conformation similar to poly(Pro)II and this structure may be important for envelope-sorting activity (Fig 7). This finding is consistent with the previous result showing that the replacement of the critical tri-Gly segment with tri-Pro did not disrupt envelope sorting [40]. However, a future experiment is needed to test whether polyGly within t75 forms an extended conformation. Together, the results presented in this work demonstrate that polyGly mediates envelope sorting by a mechanism distinct from canonical stop transfer.

Interestingly, transient expression of t75-EGFP in *N*. *benthamiana* leaves led to accumulation of the 30-kD protein in the cytosol (Fig 5). The presence of the GFP signal and the mobility on SDS-PAGE indicate that the 30-kD protein contains the entire GFP portion and lacks n75 and part but not the entire portion of c75. There are two possible scenarios regarding the synthesis of this 30-kD protein. In the first scenario, c75 prevents n75 from entering the stroma. In this case, t75-EGFP is rejected at the IEM, due to the presence of polyGly, and its N terminus including the entire n75 portion and part of c75 is removed. This scenario requires specific processing of the N terminus of c75-EGFP and protection of the remaining portion in the cytosol, which may be unprecedented. In the second scenario, c75 does not prevent n75 from entering the stroma but c75-EGFP is rejected at the IEM. Under this scenario, t75-EGFP may first be imported into the chloroplast via the general pathway and its n75 portion removed in the stroma. The resultant c75-EGFP may still stay in the chloroplast envelope surface as the 38-kD protein or processed to the 30-kD protein, which is released from the envelope to the cytosol. Interestingly, the processing activity most likely at the chloroplast IMS that removes majority but not the entire portion of c75 from the Toc75 precursor has been reported both *in vitro* [35] and *in vivo* [69]. c75-EGFP found in the envelope surface may have escaped from such processing due to its folding or localization. Addressing these possibilities should provide a detailed mechanism of polyGly-dependent protein sorting and add to our understanding of protein homeostasis in general.

## Supporting Information

S1 Table Primers used for cloning. (PDF)

S1 Fig Import of Tic22 into isolated chloroplasts followed by fractionation. Radiolabeled precursor of Tic22 was imported into isolated chloroplasts and its distribution to the total chloroplasts (T), soluble fraction (S1), peripheral membrane fraction (S2), and integral membrane fraction (P) was analyzed as described in the legend to Fig 2A. pr and m indicate precursor and mature forms, respectively. Proteins in the identical gel were visualized by phosphorimaging (top) and Coomassie Brilliant Blue staining (bottom). The experiments were done concurrently with those shown in Figs 2A and 3A.
(PDF)

S2 Fig Import of Tic22 and Tic40 into isolated chloroplasts followed by protease treatments. Radiolabeled precursors of proteins indicated at left were imported into isolated chloroplasts and their sensitivity to thermolysin (tlysin) or trypsin (tryp) with or without the presence of 1% Triton X-100 (TX) was analyzed as described in the legend to Fig 2B. Proteins were visualized by phosphorimaging. pr, i, and m indicate precursor, intermediate, and mature forms, respectively. The experiments were done concurrently with those shown in Figs 2B and 3B.
(PDF)

S3 Fig Effects of incubation time on thermolysin-susceptibility of imported proteins. Radiolabeled t75-EGFP or precursors of proteins indicated at left were imported into isolated chloroplasts for 30 min, followed by incubation with thermolysin at varying concentrations and for varying times indicated at top, and analyzed as described in Fig 2B. Proteins were visualized by phosphorimaging. For t75-EGFP, the precursor containing the full t75 portion, the intermediate lacking n75, and the 27-kD protease-resistant form are indicated as t75-EGFP, c75-EGFP, and 27, respectively. For other proteins, pr, i, and m indicate precursor, intermediate, and mature forms, respectively; DGD1 does not carry a transit peptide and the imported protein is indicated with an arrow at right.
(PDF)

S4 Fig Confocal microscopy analysis of *N*. *benthamiana* leaves transiently expressing Plsp1, t75-EGFP, or t75-EGFP_GGA_ at lower magnification. Leaves of N. benthamiana were agroinfiltrated with constructs encoding proteins shown at left and analyzed as in Fig 4. In the overlay, signals from the GFP and chlorophyll channels are depicted as green and magenta, respectively. Visualized at 1× zoom. Scale bars represent 50 μm.
(PDF)

S5 Fig Confocal microscopy analysis of *N*. *benthamiana* leaves transiently expressing t75-EGFP at higher magnification. Leaves of N. benthamiana expressing t75-EGFP visualized at 5× zoom. Scale bars represent 10 μm.
(PDF)

S6 Fig Confocal microscopy analysis of *N*. *benthamiana* leaves transiently expressing t75-EGFP_GGA_ at higher magnification. Leaves of N. benthamiana expressing t75GGA-EGFP visualized at 5× zoom. Scale bars represent 10 μm.
(PDF)

S7 Fig The primary sequences of tp75-mSS and tp75-EGFP. In both sequences, the N-terminal 35 residues corresponding to n75 are in the shaded box. In the t75-mSS sequence, residues deleted in t75?86–103 are double-underlined, the N-terminal five residues of mature psToc75 are underlined, and C-terminal six His residues derived from the pET23 vector are italicized. In the t75-EGFP sequence, the residues replaced with tri-Ala in the t75GGA-EGFP are double-underlined, the N-terminal 10 residues of mature Toc75 are underlined, and the 17 residues derived form a linker N terminus to EGFP in pB-CG are italicized.
(PDF)

S8 Fig Preparation of pB-CG. (PDF)
