# Supplementary material for: Polyglycine Acts as a Rejection Signal for Protein Transport at the Chloroplast Envelope
Source: PLoS One. 2016 Dec 9;11(12):e0167802. doi: 10.1371/journal.pone.0167802 (PMC5147994; doi:10.1371/journal.pone.0167802)
Supplement: S1 Table — (PDF) [file pone.0167802.s001.pdf]

S1 Table. Primers used for cloning.

Sequences are from 5’ to 3’. Substituted nucleotides were indicated with capital characters.

For subcloning of t75 and t75$_{\Delta 86\text{-}103}$ from pET23-pr75 to prepare pET23-t75-mSS variants	
Forward	TGCCATGGGTACTTCCGTTATTC (*Nco*I site is underlined)
Reverse	CCTCGAGCATGCATGATTTTGGTTCGTCGGCGAT (*Sph*I site is underlined)
For generation of pET23-pr75$_{\Delta 86\text{-}103}$	
For 5’-fragment encoding residues 1-85 of psToc75 and *Nde*I and *Bam*HI sites.	
Forward	CCATATGGGTACTTCCGTTATTC (*Nde*I site is underlined)
Reverse	GGGATCCAGAAAAATTAGGGA (*Bam*HI site is underlined)
For 3’-fragment encoding residues 100-125 and *Bam*HI and *Eco*RI sites.	
Forward	TTCATCTGGATCCGGAGGCGGT (*Bam*HI site is underlined)
Reverse	GGATAGAATTCTCGACCAGAACGA (*Eco*RI site is underlined)
For subcloning of t75 and t75$_{GGA}$ into pB-CG	
Forward	TAACTAGTATGCGTACTTCCGTTATTCCC (*Spe*I site is underlined)
Reverse	TAAGATCTCGAATCCCAATCTTCTGATTTTGG (*Bgl*II site is underlined)
For subcloning of t75-EGFP and t75$_{GGA}$-EGFP from pB-CG to pGEMTEasy	
Forward	TAACTAGTATGCGTACTTCCGTTATTCCC
Reverse	CGAGATCAGTTATCTAGATCCGGTGGATCC
