# Supplementary material for: Polyglycine Acts as a Rejection Signal for Protein Transport at the Chloroplast Envelope
Source: PLoS One. 2016 Dec 9;11(12):e0167802. doi: 10.1371/journal.pone.0167802 (PMC5147994; doi:10.1371/journal.pone.0167802)
Supplement: S1 Fig — Radiolabeled precursor of Tic22 was imported into isolated chloroplasts and its distribution to the total chloroplasts (T), soluble fraction (S1), peripheral membrane fraction (S2), and integral membrane fraction (P) was analyzed as described in the legend to Fig 2A. pr and m indicate precursor and mature forms, respectively. Proteins in the identical gel were visualized by phosphorimaging (top) and Coomassie Brilliant Blue staining (bottom). The experiments were done concurrently with those shown in Figs 2A and 3A. (PDF) [file pone.0167802.s002.pdf]

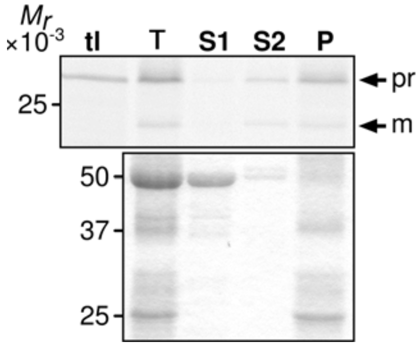

S1 Fig. Import of Tic22 into isolated chloroplasts followed by fractionation.

Radiolabeled precursor of Tic22 was imported into isolated chloroplasts and its distribution to the total chloroplasts (T), soluble fraction (S1), peripheral membrane fraction (S2), and integral membrane fraction (P) was analyzed as described in the legend to Fig 2A. pr and m indicate precursor and mature forms, respectively. Proteins in the identical gel were visualized by phosphorimaging (top) and Coomassie Brilliant Blue staining (bottom). The experiments were done concurrently with those shown in Figs 2A and 3A.
