# Supplementary material for: Polyglycine Acts as a Rejection Signal for Protein Transport at the Chloroplast Envelope
Source: PLoS One. 2016 Dec 9;11(12):e0167802. doi: 10.1371/journal.pone.0167802 (PMC5147994; doi:10.1371/journal.pone.0167802)
Supplement: S2 Fig — Radiolabeled precursors of proteins indicated at left were imported into isolated chloroplasts and their sensitivity to thermolysin (tlysin) or trypsin (tryp) with or without the presence of 1% Triton X-100 (TX) was analyzed as described in the legend to Fig 2B. Proteins were visualized by phosphorimaging. pr, i, and m indicate precursor, intermediate, and mature forms, respectively. The experiments were done concurrently with those shown in Figs 2B and 3B. (PDF) [file pone.0167802.s003.pdf]

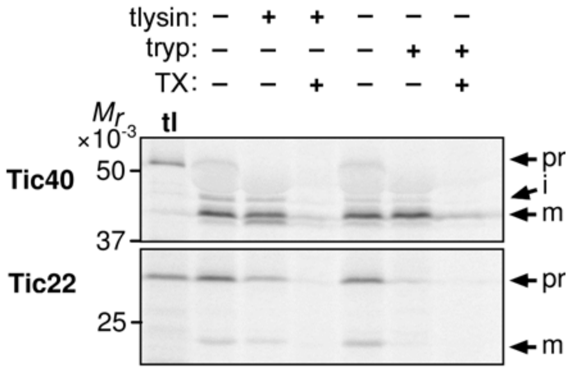

S2 Fig. Import of Tic22 and Tic40 into isolated chloroplasts followed by protease treatments. Radiolabeled precursors of proteins indicated at left were imported into isolated chloroplasts and their sensitivity to thermolysin (tlysin) or trypsin (tryp) with or without the presence of 1% Triton X-100 (TX) was analyzed as described in the legend to Fig 2B. Proteins were visualized by phosphorimaging. pr, i, and m indicate precursor, intermediate, and mature forms, respectively. The experiments were done concurrently with those shown in Figs 2B and 3B.
