# Supplementary material for: Polyglycine Acts as a Rejection Signal for Protein Transport at the Chloroplast Envelope
Source: PLoS One. 2016 Dec 9;11(12):e0167802. doi: 10.1371/journal.pone.0167802 (PMC5147994; doi:10.1371/journal.pone.0167802)
Supplement: S3 Fig — Radiolabeled t75-EGFP or precursors of proteins indicated at left were imported into isolated chloroplasts for 30 min, followed by incubation with thermolysin at varying concentrations and for varying times indicated at top, and analyzed as described in Fig 2B. Proteins were visualized by phosphorimaging. For t75-EGFP, the precursor containing the full t75 portion, the intermediate lacking n75, and the 27-kD protease-resistant form are indicated as t75-EGFP, c75-EGFP, and 27, respectively. For other proteins, pr, i, and m indicate precursor, intermediate, and mature forms, respectively; DGD1 does not carry a transit peptide and the imported protein is indicated with an arrow at right. (PDF) [file pone.0167802.s004.pdf]

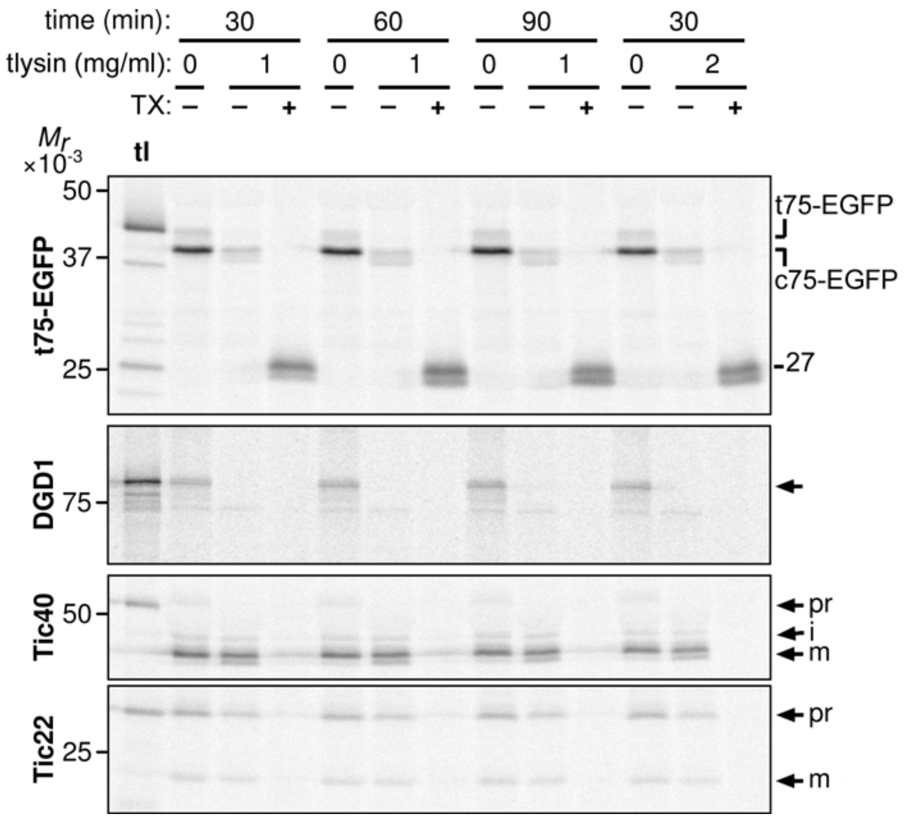

S3 Fig. Effects of incubation time on thermolysin-susceptibility of imported proteins. Radiolabeled t75-EGFP or precursors of proteins indicated at left were imported into isolated chloroplasts for 30 min, followed by incubation with thermolysin at varying concentrations and for varying times indicated at top, and analyzed as described in Fig 2B. Proteins were visualized by phosphorimaging. For t75-EGFP, the precursor containing the full t75 portion, the intermediate lacking n75, and the 27-kD protease-resistant form are indicated as t75-EGFP, c75-EGFP, and 27, respectively. For other proteins, pr, i, and m indicate precursor, intermediate, and mature forms, respectively; DGD1 does not carry a transit peptide and the imported protein is indicated with an arrow at right.
