# Supplementary material for: Polyglycine Acts as a Rejection Signal for Protein Transport at the Chloroplast Envelope
Source: PLoS One. 2016 Dec 9;11(12):e0167802. doi: 10.1371/journal.pone.0167802 (PMC5147994; doi:10.1371/journal.pone.0167802)
Supplement: S4 Fig — Leaves of N. benthamiana were agroinfiltrated with constructs encoding proteins shown at left and analyzed as in Fig 4. In the overlay, signals from the GFP and chlorophyll channels are depicted as green and magenta, respectively. Visualized at 1× zoom. Scale bars represent 50 μm. (PDF) [file pone.0167802.s005.pdf]

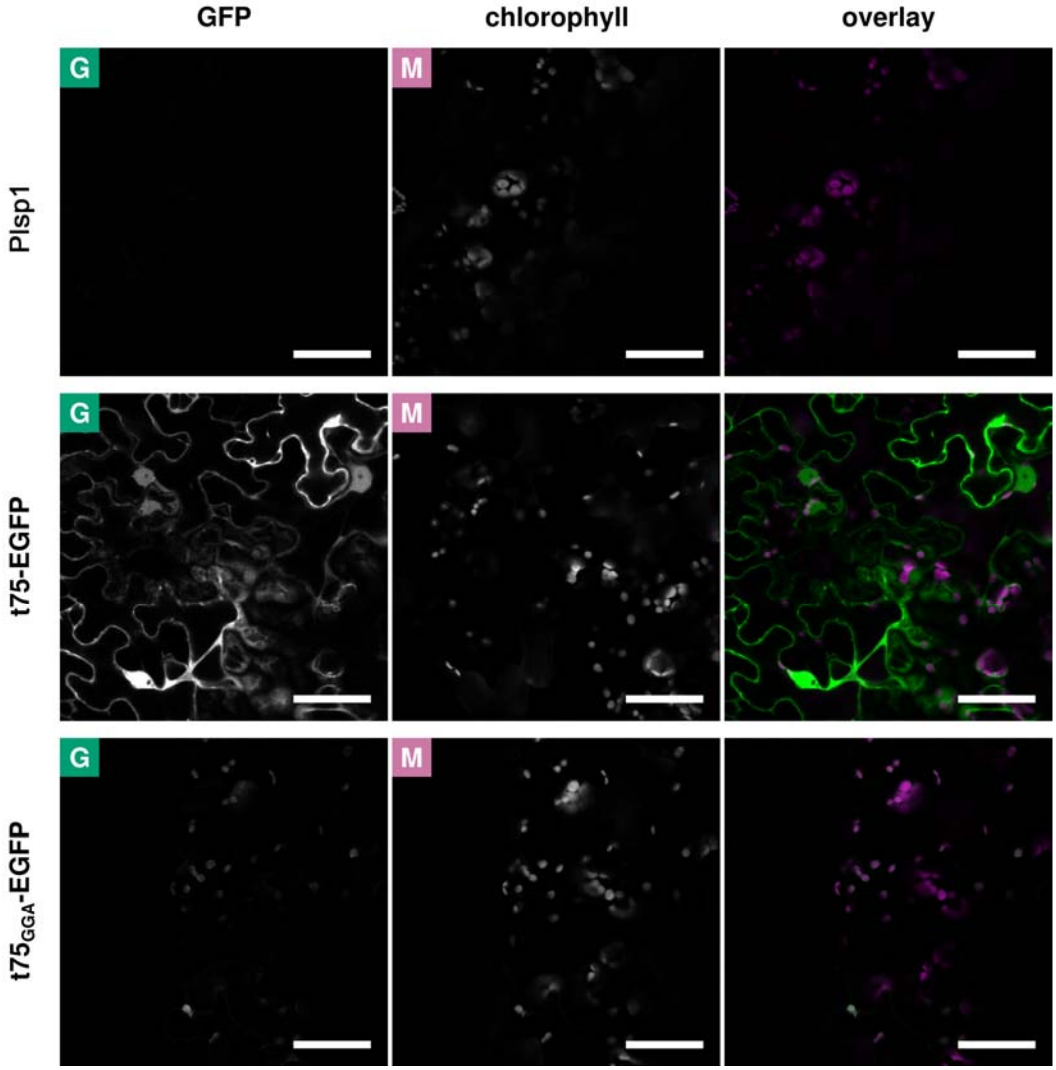

S4 Fig. Confocal microscopy analysis of *N. benthamiana* leaves transiently expressing Plsp1, t75-EGFP, or t75-EGFP$_{GGA}$ at lower magnification.

Leaves of *N. benthamiana* were agroinfiltrated with constructs encoding proteins shown at left and analyzed as in Fig 4. In the overlay, signals from the GFP and chlorophyll channels are depicted as green and magenta, respectively. Visualized at 1× zoom. Scale bars represent 50 µm.
