# Supplementary material for: Polyglycine Acts as a Rejection Signal for Protein Transport at the Chloroplast Envelope
Source: PLoS One. 2016 Dec 9;11(12):e0167802. doi: 10.1371/journal.pone.0167802 (PMC5147994; doi:10.1371/journal.pone.0167802)
Supplement: S5 Fig — Leaves of N. benthamiana expressing t75-EGFP visualized at 5× zoom. Scale bars represent 10 μm. (PDF) [file pone.0167802.s006.pdf]

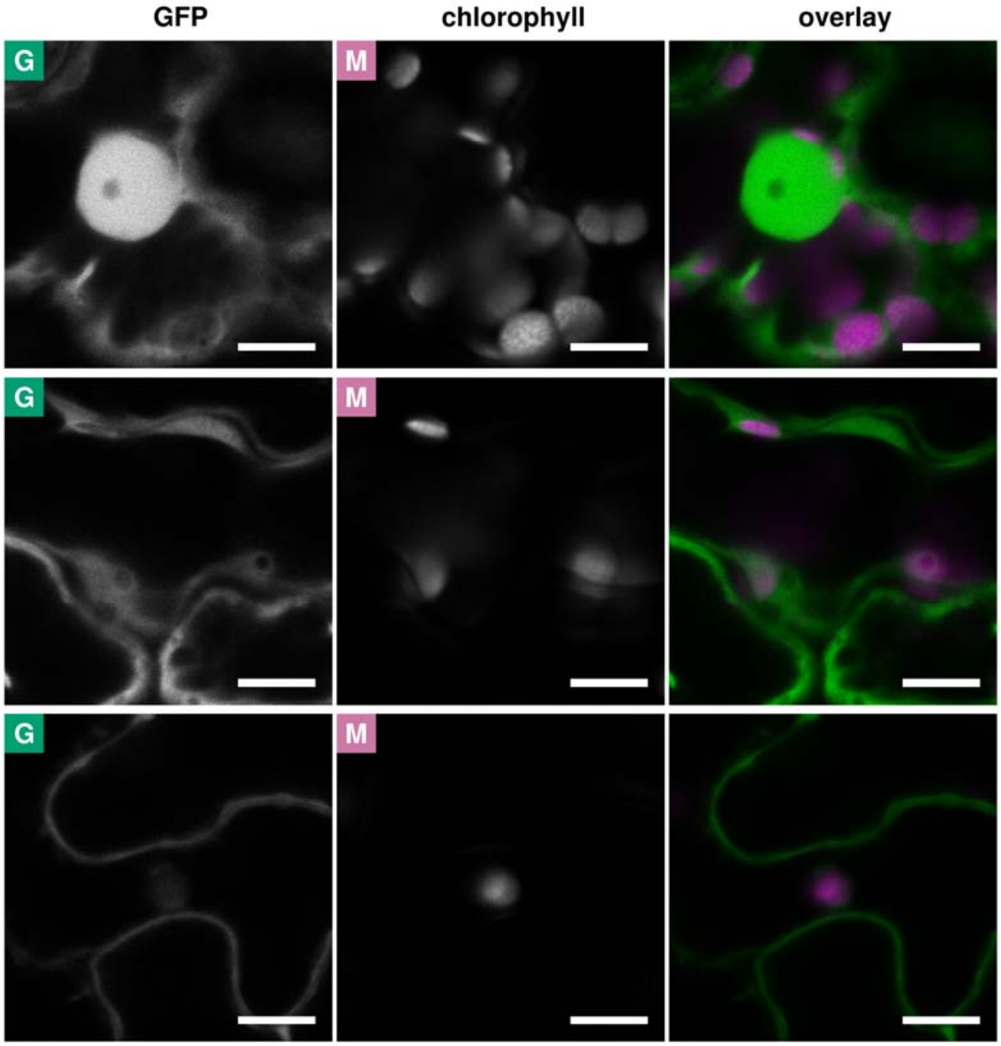

S5 Fig. Confocal microscopy analysis of *N. benthamiana* leaves transiently expressing t75-EGFP at higher magnification.

Leaves of *N. benthamiana* expressing t75-EGFP visualized at 5× zoom. Scale bars represent 10 µm.
