# Supplementary material for: Polyglycine Acts as a Rejection Signal for Protein Transport at the Chloroplast Envelope
Source: PLoS One. 2016 Dec 9;11(12):e0167802. doi: 10.1371/journal.pone.0167802 (PMC5147994; doi:10.1371/journal.pone.0167802)
Supplement: S7 Fig — In both sequences, the N-terminal 35 residues corresponding to n75 are in the shaded box. In the t75-mSS sequence, residues deleted in t75?86–103 are double-underlined, the N-terminal five residues of mature psToc75 are underlined, and C-terminal six His residues derived from the pET23 vector are italicized. In the t75-EGFP sequence, the residues replaced with tri-Ala in the t75GGA-EGFP are double-underlined, the N-terminal 10 residues of mature Toc75 are underlined, and the 17 residues derived form a linker N terminus to EGFP in pB-CG are italicized. (PDF) [file pone.0167802.s008.pdf]

>t75-mSS

MGTSVIPNRL TPTLTTHPSR RRNDHITTRT SSLKCHLSPS SGDNNDSFNS SLLKTISTTV- 60
AVSSAAASAF FLTGSLHSPF PNFSGLNAAA GGGAGGGGGG SSSSGGGGGG WFNGDEGSFW-120
SRILSPARAI ADEPKSCMQV WPPIGKKKFE TLSYLPPLTR DQLLKEVEYL LRKGWVPCLE-180
FELEKGFVYR EHNKSPGYYD GRYWTMWKLP MFGTTDASQV LKELDEVVAA YPQAFVRIIG-240
FDNVRQVQCI SFIAHTPESY LE*HHHHHH*-278

>t75-EGFP

MRTSVIPNRL TPTLTTHPSR RRNDHITTRT SSLKCHLSPS SGDNNDSFNS SLLKTISTTV- 60
AVSSAAASAF FLTGSLHSPF PNFSGLNAAA GGGAGGGGGG SSSSGGGGGG WFNGDEGSFW-120
SRILSPARAI A**D**EPKSEDWD *SRSAAAAAAA AAAGPVAT*MV SKGEELFTGV VPILVELDGD-180
VNGHKFSVSG EGEGDATYGK LTLKFICTTG KLPVPWPTLV TTLTYGVQCF SRYPDHMKQH-240
DFFKSAMPEG YVQERTIFFK DDGNYKTRAE VKFEGDTLVN RIELKGIDFK EDGNILGHKL-300
EYNYNSHNVY IMADKQKNGI KVNFKIRHNI EDGSVQLADH YQQNTPIGDG PVLLPDNHYL-360
STQSALSKDP NEKRDHMVLL EFVTAAGITL GMDELYK-397

S7 Fig. The primary sequences of t75-mSS and t75-EGFP variants.

In both sequences, the N-terminal 35 residues corresponding to n75 are in the shaded box. In the t75-mSS sequence, residues deleted in $t75_{\Delta 86\text{-}103}$ are double-underlined, the N-terminal five residues of mature psToc75 are underlined, and C-terminal six His residues derived from the pET23 vector are italicized. In the t75-EGFP sequence, the residues replaced with tri-Ala in the $t75_{GGA}$-EGFP are double-underlined, the N-terminal 10 residues of mature Toc75 are underlined, and the 17 residues derived form a linker N terminus to EGFP in pB-CG are italicized.
