# Supplementary material for: Polyglycine Acts as a Rejection Signal for Protein Transport at the Chloroplast Envelope
Source: PLoS One. 2016 Dec 9;11(12):e0167802. doi: 10.1371/journal.pone.0167802 (PMC5147994; doi:10.1371/journal.pone.0167802)
Supplement: S8 Fig — (PDF) [file pone.0167802.s009.pdf]

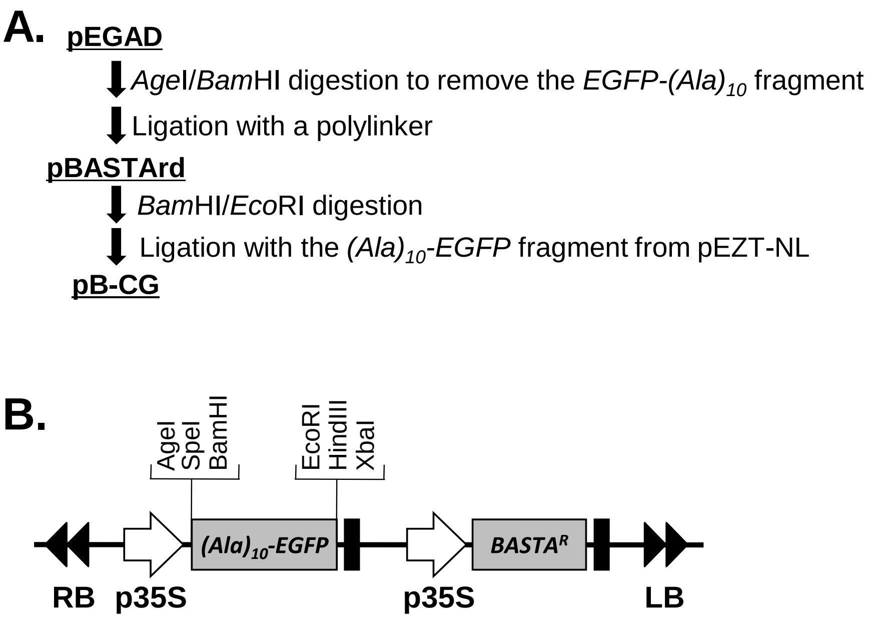

S8 Fig. Preparation of pB-CG.

(a) A schematic showing the construction of pB-CG. The fragment encoding EGFP-$(Ala)_{10}$ in pEGAD plasmid [Cutler, S. R., Ehrhardt, D. W., Griffitts, J. S. and Somerville, C. R. (2000) Random GFP::cDNA fusions enable visualization of subcellular structures in cells of Arabidopsis at a high frequency. Proc. Natl. Acad. Sci. U S A. **97**, 3718-3723] was removed by digestion with *Age*I and *Bam*HI followed by ligation to a polylinker cassette made of two oligonucleotides with a *Bam*HI site (double underlined) (5'-CCGGTACTAGTGGATCCCCCGGGGAATTCAAGCTTA-3' and 5'-GATCTAAGCTTGAATTCCCCGGGGGATCCACTAGTA-3'), yielding pBASTArd. During this ligation, the *Bam*HI site (GGATCC) 3' to the EGFP-$(Ala)_{10}$ coding sequence in pEGAD was replaced by GGATCT. A sequence encoding $(Ala)_{10}$-EGFP was amplified by PCR using pEZT-NL (http://deepgreen.stanford.edu/) and the following set of primers (the forward primer including a *Bam*HI site as underlined: 5'-AAGGATCCGCTGCTGCCGCTGCCGCTGCGGCAGCGGCCGGACCGGTCGCCACCATG-3'; and the reverse primer including *Eco*RI site as underlined 5'-AAGAATTCTTACTTGTACAGCTCGTCCATGCC-3'). The resultant fragment was subcloned into pBASTArd using the *Bam*HI and *Eco*RI sites, yielding pB-CG.

(b) The T-DNA region of pB-CG. Right and left borders (RB and LB), CaMV35S promoters (p35S), the basta herbicide resistance locus (BASTA[R]), and the terminator (black box), all of which originated from pEGAD, are indicated.
